# Circulating Noncoding RNAs as Candidate Biomarkers of Endocrine and Metabolic Diseases

**DOI:** 10.1155/2018/9514927

**Published:** 2018-07-17

**Authors:** Guido Sebastiani, Claudiane Guay, Mathieu Latreille

**Affiliations:** ^1^Department of Medicine, Surgery and Neurosciences, University of Siena, Siena, Italy; ^2^Fondazione Umberto di Mario, Toscana Life Sciences, Siena, Italy; ^3^Department of Fundamental Neurosciences, University of Lausanne, Lausanne, Switzerland; ^4^MRC London Institute of Medical Sciences, Du Cane Road, London W12 0NN, UK; ^5^Institute of Clinical Sciences, Faculty of Medicine, Imperial College London, Du Cane Road, London W12 0NN, UK

Over the last decade, several studies focused on circulating nucleic acids as potential cell-cell communication mediators or as disease biomarkers, thus potentially improving and extending the strategies to understand and diagnose/prognose many different diseases. Particularly, noncoding RNAs (including microRNAs, piRNAs, and long noncoding RNAs) received a bulk of attention due to their role as intracellular regulators of gene expression. Most of the efforts have focused on microRNAs which are single-stranded small noncoding RNAs of 19–24 nucleotides in length that negatively regulate gene expression through their binding to the 3′ untranslated region (UTR) of the messenger RNA (mRNA) target. MicroRNAs have been demonstrated to be part of pivotal cellular processes through their ability to posttranscriptionally modulate expression levels of many target genes; consequently, they were associated to the regulation of important metabolic processes ranging from insulin secretion (pancreatic islet) and insulin sensitivity (muscle, adipose tissue) to bone deposition and absorption.

It is now well known that microRNAs can be secreted by virtually every cell type and deploy important functions in cell-cell and interorgan communication, being found in most of the extracellular fluids through the associations with several circulating components including Argonaute proteins, HDL particles, microvesicles, and exosomes.

In this Special Issue, authors focused on different aspects of circulating microRNAs' biology and function in endocrine and metabolic diseases as well as addressed issues that needs further clarification. M. A. C. Pomatto et al. described the role of extracellular vesicles (EVs) in the transport of circulating noncoding RNAs and their role in several diseases including metabolic syndrome, diabetes, thyroid disorders, preeclampsia, and atherosclerosis. They also discuss the challenges associated with EV isolation from different biological fluids (serum, plasma, and cell culture media) and content analysis which could explain the poor technical and data reproducibility observed between different research groups. The importance of EVs in noncoding RNA transport and interorgan communication has been elegantly shown by Thomou et al. whereby circulating EVs derived from adipose tissue modulate hepatic function/metabolism. Further addressing the role of microRNA networks in adipose tissue may help to clarify the entire mechanism by which adipose tissue regulates the activity of distant organs. To this aim, Y. O. N. Lopez et al. elucidated the regulatory network existing between adipose tissue miR-24, miR-146a, and miR-30d and the antiangiogenic factor secreted frizzled-related protein 4 (SFRP4) in obesity and type 2 diabetes (T2D). These microRNAs were found to be upregulated in type 2 diabetes (T2D) and obese patients compared to healthy controls and correlated with elevated whole body adiposity and with SFRP4 levels in visceral adipose tissue, thus supporting the presence of a regulatory loop between microRNAs and SFRP4 in obesity and T2D pathophysiology.

Deciphering interorgan communication by EVs may also represent a novel pathological component of different diseases as observed in gestational diabetes mellitus (GDM) in which circulating microRNAs derived from placenta EVs may trigger insulin resistance and beta-cell dysfunction. Toward this end, E. Guarino et al. now reports on most recent findings on exosomes and circulating noncoding RNAs in GDM and discuss how technical development may improve the identification and characterization of placenta-derived circulating exosomes.

Additionally, microRNAs have been shown to modulate vascular endothelium homeostasis and bone metabolism. F. Barutta et al. and M. Materozzi et al. reviewed the current knowledge on the pathological effect of noncoding RNAs in microvascular complications in diabetes and bone osteoporotic alterations, respectively. Whereas alteration in microRNA activity may play a crucial role in vascular complications found in diabetics, these tiny regulatory circulating molecules may represent potential biomarkers of bone activity as well.

Finally, several ongoing studies are trying to characterize the potential use of circulating miRNAs as biomarkers of therapeutic response, in order to anticipate the final outcome. In this special issue, G. Catanzaro et al. explored such possibility by analyzing circulating miRNAs in plasma of T2D patients before and after dipeptil dipeptidase IV inhibitor (DPP-IVi) sitagliptin therapy. Analysis of circulating microRNA profiles revealed three microRNAs correlating with responses to DPP-IVi in those patients: miR-378 which was associated with a resistance to DPP-IVi and miR-126-3p and miR-223 which were positively correlated with DPP-IVi response in T2D patients. This study highlights the potential usefulness of circulating microRNAs in predicting the response of patients to glucose-lowering drugs used and may help contribute to improving personalized medicine in the near future.

*Guido Sebastiani* *Claudiane Guay* *Mathieu Latreille*

